# AC Electromagnetic Field Controls the Biofilms on the Glass Surface by *Escherichia coli* & *Staphylococcus epidermidis* Inhibition Effect

**DOI:** 10.3390/ma16217051

**Published:** 2023-11-06

**Authors:** Natsu Aoyama, Hideyuki Kanematsu, Dana M. Barry, Hidekazu Miura, Akiko Ogawa, Takeshi Kogo, Risa Kawai, Takeshi Hagio, Nobumitsu Hirai, Takehito Kato, Michiko Yoshitake, Ryoichi Ichino

**Affiliations:** 1 Department of Materials Science and Engineering, National Institute of Technology (KOSEN), Suzuka College, (Currently Asahi Kasei Co.), Suzuka 510-0294, Japan; aoyama.nd@om.asahi-kasei.co.jp (N.A.); kougo@mse.suzuka-ct.ac.jp (T.K.); kawai-r@mse.suzuka-ct.ac.jp (R.K.); 2 Research Collaboration Promotion Center, National Institute of Technology (KOSEN), Suzuka College, Suzuka 510-0294, Japan; 3 Department of Electrical and Computer Engineering, Clarkson University, Potsdam, NY 13699, USA; dmbarry@clarkson.edu; 4 Faculty of Medical Engineering, Suzuka University of Medical Science, Suzuka 510-0293, Japan; miura-h@suzuka-u.ac.jp; 5 Department of Chemistry and Biochemistry, National Institute of Technology (KOSEN), Suzuka College, Suzuka 510-0294, Japan; ogawa@chem.suzuka-ct.ac.jp (A.O.); hirai@chem.suzuka-ct.ac.jp (N.H.); 6 Institutes of Innovation for Future Society, Graduate School of Engineering, Nagoya University, Nagoya 464-8601, Japan; hagio@mirai.nagoya-u.ac.jp; 7 National Institute of Technology (KOSEN), Oyama College, Oyama 323-0806, Japan; kato_t@oyama-ct.ac.jp; 8 National Institute for Materials Science (NIMS), Tsukuba 305-0047, Japan; yoshitake.michiko@nims.go.jp; 9 Graduate School of Engineering Chemical Systems Engineering 2, Graduate School of Engineering, Nagoya University, Nagoya 464-8601, Japan; ichino@numse.nagoya-u.ac.jp

**Keywords:** biofilms, electromagnetic field, *Escherichia coli*, *Staphylococcus epidermidis*

## Abstract

Biofilms, mainly comprised of bacteria, form on materials’ surfaces due to bacterial activity. They are generally composed of water, extracellular polymeric substances (polysaccharides, proteins, nucleic acids, and lipids), and bacteria. Some bacteria that form biofilms cause periodontal disease, corrosion of the metal materials that make up drains, and slippage. Inside of a biofilm is an environment conducive to the growth and propagation of bacteria. Problems with biofilms include the inability of disinfectants and antibiotics to act on them. Therefore, we have investigated the potential application of alternating electromagnetic fields for biofilm control. We obtained exciting results using various materials’ specimens and frequency conditions. Through these studies, we gradually understood that the combination of the type of bacteria, the kind of material, and the application of an electromagnetic field with various low frequencies (4 kHz–12 kHz) changes the circumstances of the onset of the biofilm suppression effect. In this study, relatively high frequencies (20 and 30 kHz) were applied to biofilms caused by *Escherichia coli (E. coli)* and *Staphylococcus epidermidis* (*S. epidermidis*), and quantitative evaluation was performed using staining methods. The sample surfaces were analyzed by Raman spectroscopy using a Laser Raman spectrometer to confirm the presence of biofilms on the surface.

## 1. Introduction

Biofilms are slimy structures formed when bacteria release extracellular polymeric substances (EPS) on their exterior and cover themselves with a film-like substance. Biofilms are observed in the growth environment of microbial cells, which can grow in moderate temperatures and humidity, and form in almost all environments on Earth’s surfaces [1,2,3,4,5,6,7,8]. The inside of a biofilm is an environment where bacteria can quickly multiply and grow. Most biofilms are formed on solid surfaces, but they can also exist as dispersed phases in liquids when they form at the gas–liquid interface [9,10,11,12,13,14,15,16,17,18].

The inside of the biofilm structure is an environment in which bacteria can easily multiply and grow. Therefore, biofilm formation causes various problems in our daily lives and for industries. Examples include infections and chronic diseases in the medical field, where biofilms are formed when indigenous bacteria adhere to urinary catheters and stents. This causes a serious problem of immune suppression and infection. Recently, it has been reported that biofilms are responsible for approximately 80% of infections caused by bacterial cells in vivo [19,20,21,22,23,24,25,26,27,28]. In the industrial field, biofilms cause corrosion and clogging of the metal materials that make up drainage channels. They also cause slime that adheres to the glass surface on the inside of water tanks. Biofilms are not easily affected by disinfectants and antibiotics. This is most likely because biofilm constituents block their penetration. Nutrients, including inorganic substances, some organic substances, and small molecules, are taken in from outside of the biofilm to provide nutrients to sustain life. The internal structure of the biofilm has “open water channels” which allow a relatively free exchange of materials through the water that makes up most of the biofilm [29,30,31,32,33]. This internal state ensures the life support of biofilm cells and supports the growth of the biofilm from the inside. Although biofilms can be effectively utilized for sewage treatment, toxic waste disposal, and other purposes, they must be controlled in most cases. Mechanical removal of biofilms is difficult for a variety of reasons.

Various methods have been proposed to control biofilms, and these have been divided into different categories. The control of biofilms with chemicals has been the most studied area in terms of water treatment [34], infection control [35], and improvement of sanitation issues [36]. Although chemical control is of course effective, it also has the aspect of always having to consider the negative effects on the environment and on people. Modification of materials (replacement with appropriate materials, alloying, surface treatment, etc.) is also a useful method. However, this method requires steps to decommission and replace existing systems. This is often difficult from a practical standpoint. It involves cost-related issues and the cessation of actual operations. Methods that do not require major modifications to existing systems, such as the introduction of chemicals into the aquatic environment, and that have a low environmental impact, can be expected to have a positive effect on these existing measures. One of these methods is the use of AC electromagnetic fields, as well as the utilization of ultraviolet lights. In fact, the application of electromagnetic fields to water treatment is a method that has been actively explored in the past [37]. The main purpose was to reduce scale-buildup in water treatment plants and piping. However, there were many contingent factors that led numerous researchers and engineers to withdraw from the application of magnetic fields, as the effects were sometimes remarkable and in other cases not so good. One reason for this may be that the concept of biofilms had not been proposed yet. As the world was entering the 21st century, the concept of biofilms came to be widely known. As one of Japan’s national projects, the authors decided to try a new application of AC electromagnetic fields and incorporating this concept [38]. The device has been put into practical use and commercialized. In these investigations, we discretely applied 4 kHz to 12 or 16 kHz to the water system. At some frequencies, the obvious control effects appeared, even though positive effects could not be seen at other frequencies. The results of these efforts have led to rights and product development, and have achieved a certain degree of success. One problem is that the mechanism has not yet been clarified. Another problem is how to easily select the frequency at which the suppression effect can be obtained in practical use, considering the results are obtained at discrete frequencies.

One of the study’s authors was involved in a project for biofilm quantification and its international standardization, and formed a committee with the SIAA (the Society of International Sustaining Growth for Antimicrobial Articles). The committee has been holding regular review meetings since 2012 to discuss biofilm quantification in general. At the meeting, one of the members suggested that the frequency of the electromagnetic field should be raised a little more. He informed us that a Japanese company had successfully commercialized a product using a high frequency for another purpose (rice cooking), not biofilm suppression [39]. In this system, the entire water system is moved by the application of high frequency, and there is a possibility that a mechanical shock can be given to the material. The frequency range was several tens of kHz. In this experiment, 20 kHz and 30 kHz were selected as the frequencies that are not too far from the available frequency range in the previous studies. They can be expected to have a wave effect on the above solution. A comparison was made with the previous results at several kilohertz to determine whether biofilm suppression would be effective in the above frequency range. The results were compared with previous results at several kilohertz.

## 2. Materials and Methods

### 2.1. Sample Preparation

**Figure 1:**
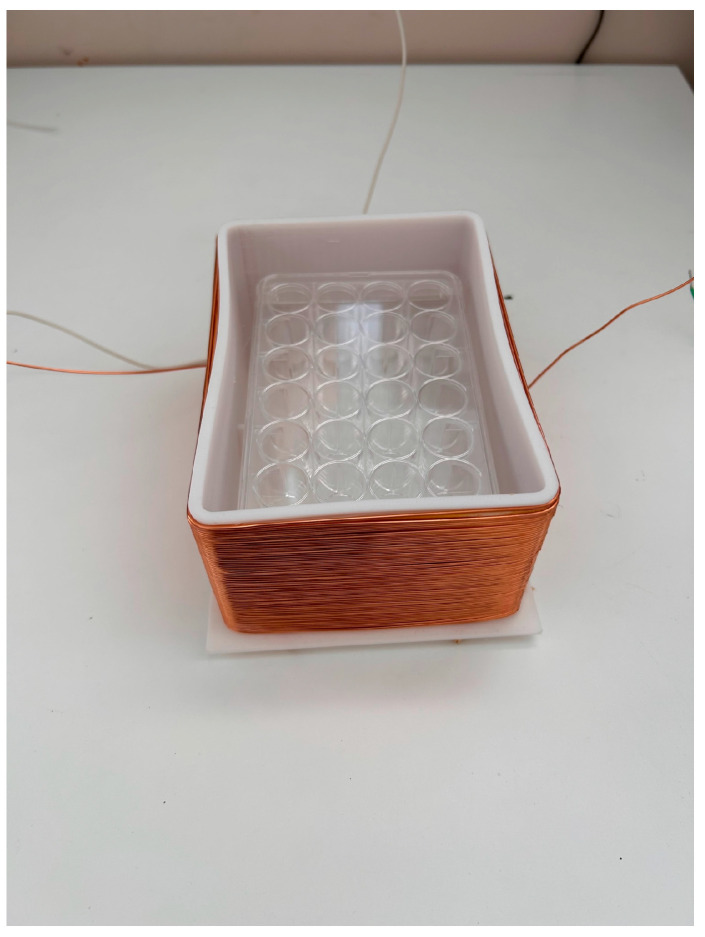
An electromagnetic coil box and a well.

**Figure 2:**
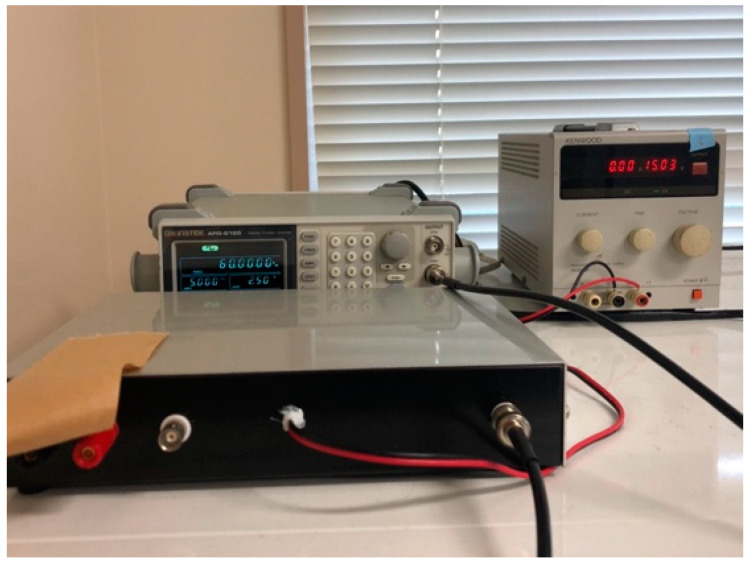
The control system of experimental apparatus.

A 1.0 × 1.0 cm^2^ glass substrate was autoclaved at 121 °C for 15 min under pressure and sterilized to form biofilms. The bacteria used were *Escherichia coli* (*E. coli*, K-12, G6) and *Staphylococcus. epidermidis* (*S. epidermidis*, ATCC35984). *E. coli* and *S. epidermidis* were pre-cultured in LB liquid medium (2068-75, M9T2881, Nakalai Tesque Corporation, Kyoto, Japan) and HI liquid medium (238400, 4210555, Becton, Dickinson, and Company, Franklin Lakes, NJ, USA), respectively, for 24 h at 37 °C with shaking. At that point, the bacteria became full-grown, with a concentration of approximately 10^9^ CFU/mL. The bacteria solution (undiluted) and the dilutions of the bacteria solution (undiluted) were 2-, 4-, and 10-fold, respectively. Two sterilized 24-well wells (MS-80240, Adhesive Cell Culture Plate 24F Independent Well Type) containing 15 glass substrates (10 mm × 10 mm, thickness: 0.9–1.2 mm) were prepared, and 2.0 mL of the prepared bacterial solution was injected into each well. As an AC electromagnetic field generator, a coil was fabricated by winding a conductor with a diameter of 0.5 mm (Figure 1) and adjusting the magnetic flux density to 1.0 mT when a current of 1.0 A flowed through the coil. One of the wells (24 plastic wells: 128 mm × 85 mm) was placed in a box (148 mm × 100 mm), and a coil of copper was wound around it (125 windings), as shown in Figure 1. The coil was connected to a constant power supply (PA 36-3A, Kenwood, Tokyo, Japan), a function generator (AFG-2125, GW Instek, Taiwan) and a constant current device (made by the authors), as shown in Figure 2. The signal source was a square wave that was introduced into the LCR resonance circuit, and the wave applied to the system was a sine wave as a result. Therefore, the magnetic field waveform was also sinusoidal. The current and voltage were tested within several tens of micro tesla, where the maximum magnetic flux density injected into the system was under 0.1 mT. The same condition and the same waveform were applied to both bacteria. The other well was placed at the same temperature, unaffected by the AC electromagnetic field, and a control experiment was conducted. The detailed conditions are shown in Table 1. The sample to which an AC electromagnetic field was applied is denoted as EM, and the sample to which no AC electromagnetic field was applied is denoted as nEM. There were three experimental samples performed under each condition (*n* = 3). The quantitative evaluation by staining shown below is an analysis of the results of the three tests, and the Raman spectroscopy test used as a qualitative test is the result of one representative of the three results. The specific methods of these tests are described in detail below.

Table 1 Experimental Condition.

### 2.2. Freeze-Drying Treatment

When the bacteria solution in each well was removed after incubation under the conditions shown in Table 1, the biofilm-formed glass sample lost water over time, making it difficult to maintain the shape of the biofilm on the glass sample. Therefore, freeze-drying was used in this experiment to maintain the shape of the biofilm and to analyze the components attached to the sample more precisely when performing Raman spectroscopic analysis.

Ethyl alcohol (C_2_H_5_OH, 99.5%, Special Grade, Wako Pure Chemical Industries, Ltd., Tokyo, Japan) was used, and 2.0 mL of distilled water: ethanol solution (7:3, 5:5, 3:7, 2:8, 1:9, 0.5:9.5, 0.2:9.8) was injected in the wells in that order. The water in the biofilm was replaced entirely by ethyl alcohol, a little at a time.

The ethyl alcohol component in the biofilm of the sample treated in the previous step was replaced with t-butyl alcohol (C_4_H_10_O, Special Grade, Kishida Chemical Co., Osaka, Japan), Ethyl alcohol and t-butyl alcohol solutions of 7:3, 5:5, 3:7, and 0:10 were injected in 2.0 mL wells in that order, immersed for 15 min, and then removed. After replacement, samples immersed in t-butyl alcohol solution were frozen in a refrigerator and evaluated.

Freeze-drying biofilms were evaluated qualitatively by Raman spectroscopy and quantitatively by crystal violet staining. Based on the results of these two evaluation methods, we evaluated the suppression effect of AC electromagnetic fields on biofilm formation. The two biofilm evaluation methods are shown below.

### 2.3. Raman Spectroscopic Analysis

The Raman spectrometer (NRS-3100, JASCO Co., Ltd., Tokyo, Japan) irradiates laser light and uses the reflected light to analyze substances’ qualitative and structural properties. The reflected and scattered light includes Raman scattered light, which is scattered at a wavelength different from that of the incident light. The bonding between molecules generates this Raman scattering light, and the mechanism is to obtain information on the substance by measuring the change in wavelength and intensity of Raman scattering. Various information about the substance can be obtained from the analyzed spectrum. The Raman shift is intrinsic to molecular bonding and can tell a substance’s composition. The peak intensity gives information on the substance’s polarization rate, orientation, and abundance. Combined with this information, it can be used for qualitative and structural analysis of the measured substance. Since biofilms comprise EPS-containing proteins, polysaccharides, etc., this analysis method can identify the composition and structure of substances adhering to the sample surface, and thus determine the present substances [40,41,42,43,44,45,46]. Although quantitative analysis is also possible based on comparison of peak values obtained by Raman spectroscopy, in this study, Raman was measured by randomly selecting areas where biofilm formation was confirmed by microscopy, so the results are used only as one qualitative result representing the presence and composition, not the entire sample surface. The sample was irradiated with laser light (532 nm), and the Raman spectra were measured under the following conditions: exposure time 1.0 s, number of integrations 3 times, measurement range 500~3500 cm^−1^, and grating 1800 L/mm.

### 2.4. Crystal Violet Staining Method

After incubation under the conditions shown in Table 1, the bacterial fluid in each well was immediately removed with a dropper. A 0.1% crystal violet solution was prepared using crystal violet (C_25_N_3_H_30_Cl, special grade, Wako Pure Chemical Industries, Ltd.). After 30 min of staining, the samples were rinsed three times with distilled water to remove excess dye adhering to the samples, and allowed to dry spontaneously for 24 h to remove residual water droplets. The crystal violet used as a reagent is positively charged and is considered to adsorb efficiently on polymers containing polysaccharides, some of the biofilms’ components. When stained with crystal violet, the stain easily identifies Gram-positive bacteria, but Gram-negative bacteria are difficult to stain. The difference in stainability is due to the thickness of the peptidoglycan layer, which is the bacteria’s cell wall. Gram-positive bacteria have a thicker peptidoglycan layer, which allows them to retain adsorbed crystal violet. In contrast, Gram-negative bacteria have a thinner peptidoglycan layer, which does not allow them to retain adsorbed crystal violet, resulting in differences in staining. Since *E. coli* is a Gram-negative bacterium and *S. epidermidis* is a Gram-positive bacterium, we considered it necessary to compare these two types of bacteria.

**Figure 3:**
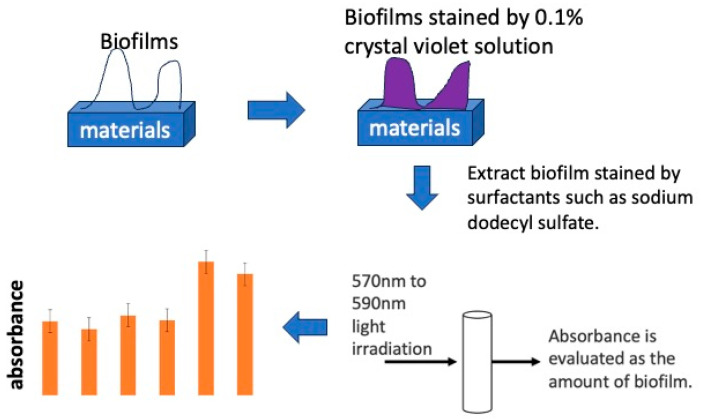
The process of biofilm quantification using staining by crystal violet solution.

The stained sample was placed in a centrifuge tube filled with 3.0 mL of a 10% sodium dodecyl sulfate solution (CH_3_(CH_2_)_11_OSO_3_Na, Wako I grade) to extract crystal violet specifically adsorbed on the sample surface. An amount of 0.20 mL of the extract was injected into 96-well wells and irradiated with 595 nm wavelength light using a plate reader (MULTISKAN FC, Thermo Scientific, Inc., Waltham, MA, USA) to measure absorbance. The absorbance obtained was evaluated as the amount of biofilm formation. A schematic diagram of the absorbance measurement is shown in Figure 3.

## 3. Results

### 3.1. Raman Spectroscopy

**Figure 4:**
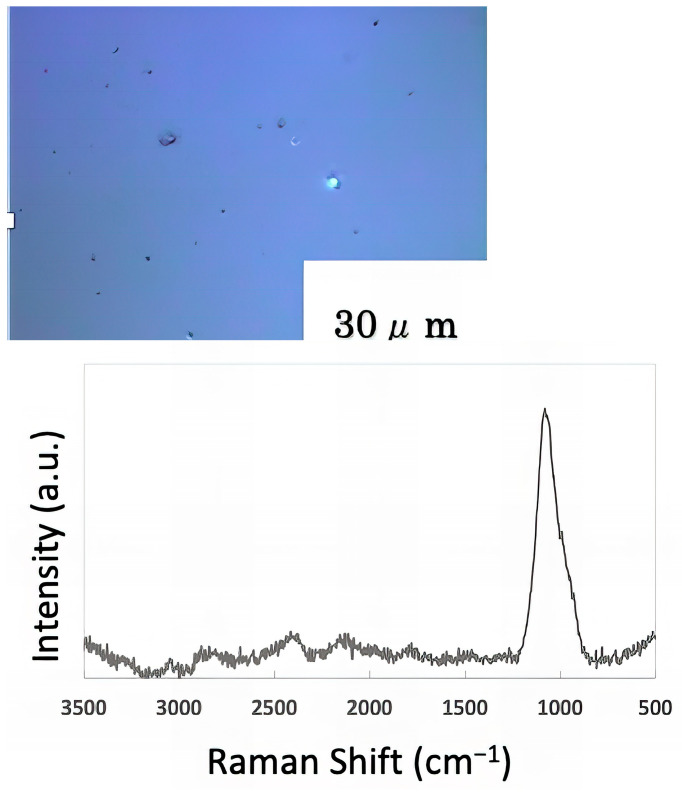
Surface image and Raman spectrum of the sample before the experiment.

**Figure 5:**
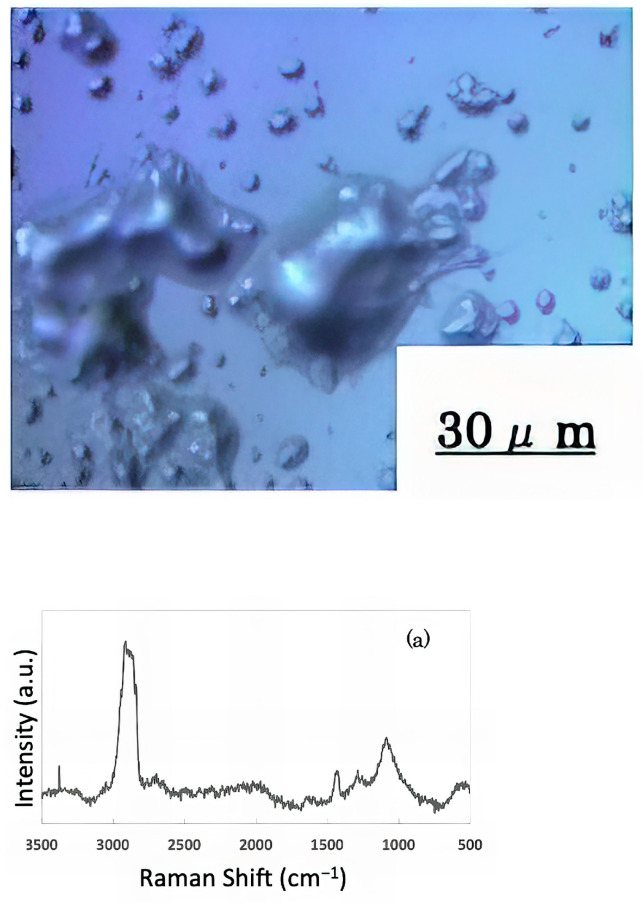

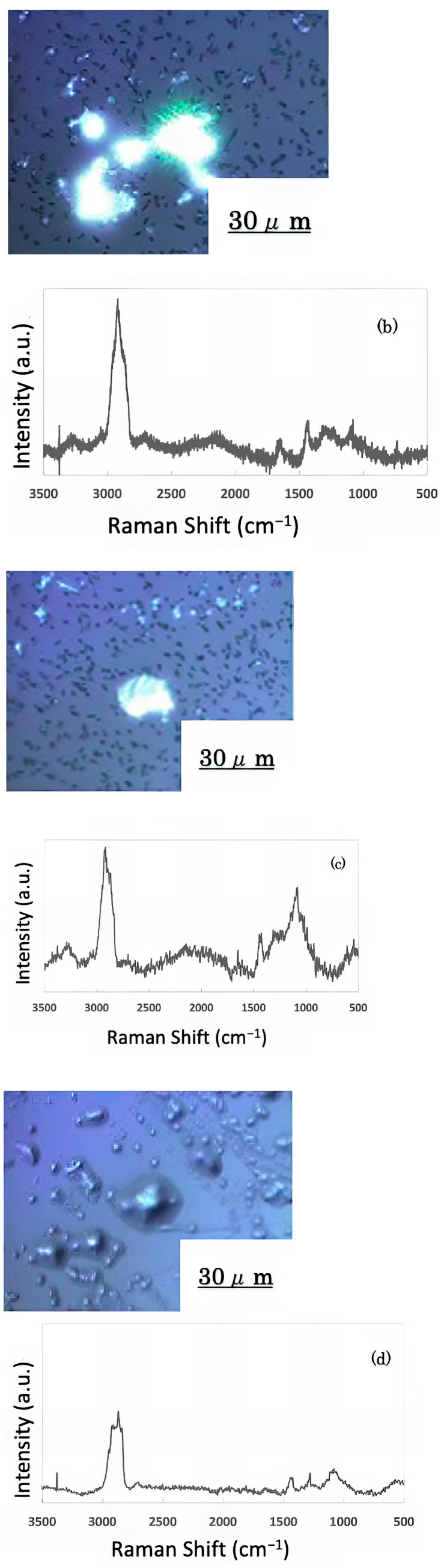
Surface image and Raman spectrum of biofilm caused by *Escherichia coli* (**a**) 20 kHz nEM, (**b**) 20 kHz EM, (**c**) 30 kHz nEM, (**d**) 30 kHz EM.

**Figure 6:**
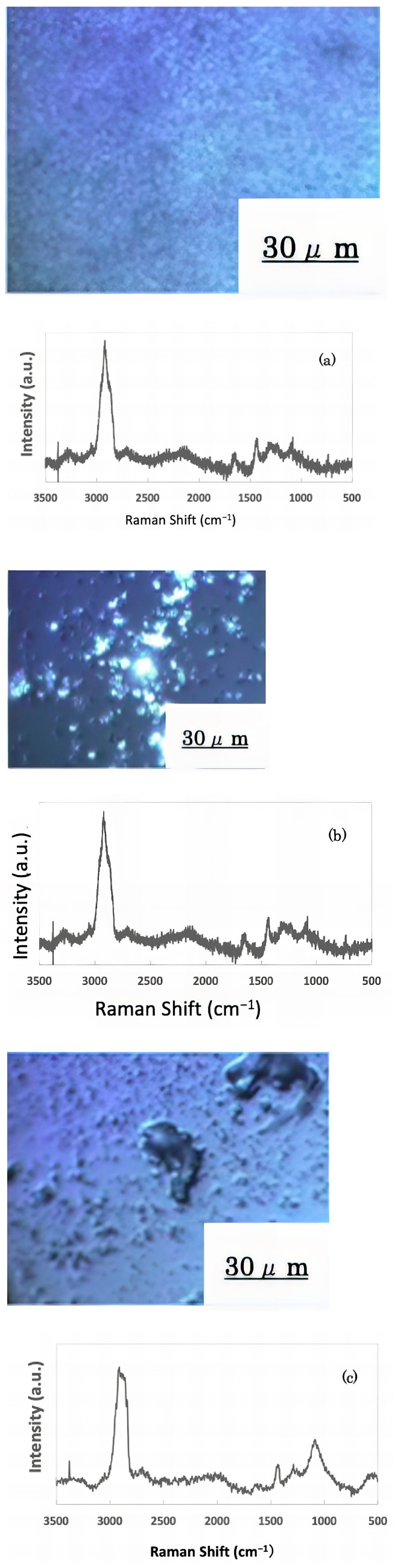

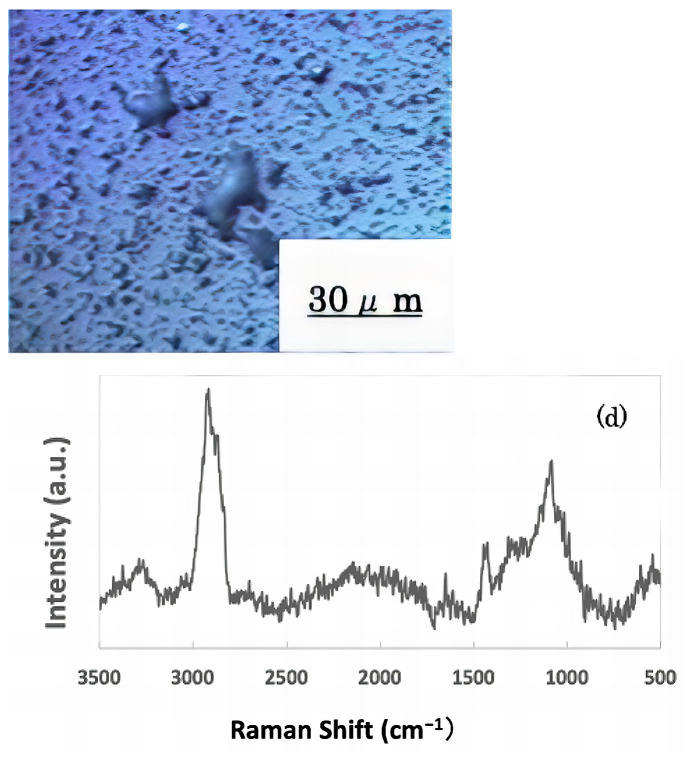
Biofilm surface image and Raman spectrum of *Staphylococcus epidermidis* (**a**) 20 kHz nEM, (**b**) 20 kHz EMm, (**c**) 30 kHz nEM, (**d**) 30 kHz EM.

The freeze-dried samples prepared in the previous step were analyzed by Raman spectroscopy to confirm whether the deposits on the glass sample surface were biofilms. The pre-experiment samples’ surface images and Raman spectra are shown in Figure 4, and surface images and Raman spectra of nEM and EM samples with *E. coli* and *S. epidermidis* are shown in Figure 5 and Figure 6. The surface of the glass plate as received was generally very flat, as seen in Figure 4. From Figure 4, we could assign only one remarkable peak around 1000 cm^−1^ to that of substrate. Therefore, in Figure 5 and Figure 6, peaks observed around 1000 cm^−1^ were confirmed as that of substrate glasses. These were the peaks of the glass used as the substrate. Some adhesion-like materials were observed in places. When a laser beam was irradiated to a localized area centered on this region, a characteristic Raman shift was observed. In Figure 5 and Figure 6, two peaks in particular were clearly observed. One of them appeared at 2928 cm^−1^ and the other one at 1440 cm^−1^~1445 cm^−1^, respectively. The peak at 2928 cm^−1^ could be assigned to lipid [47], and that at 1440 cm^−1^ also to lipid [47,48] or protein [49]. For some specimens (Figure 4) peaks around 1300 cm^−1^ were observed. These could be attributed to protein [49]. These protein and lipid peaks are part of the biofilm components and were not detected in the Raman spectra of the pre-experiment samples (Figure 4). Therefore, the presence of biofilm is indeed observed in the post-test sample. However, the differences between specimens with the application of AC electromagnetic fields and those without them were not clear.

### 3.2. Crystal Violet Staining Evaluation

#### 3.2.1. Biofilm by *E. coli*

**Figure 7:**
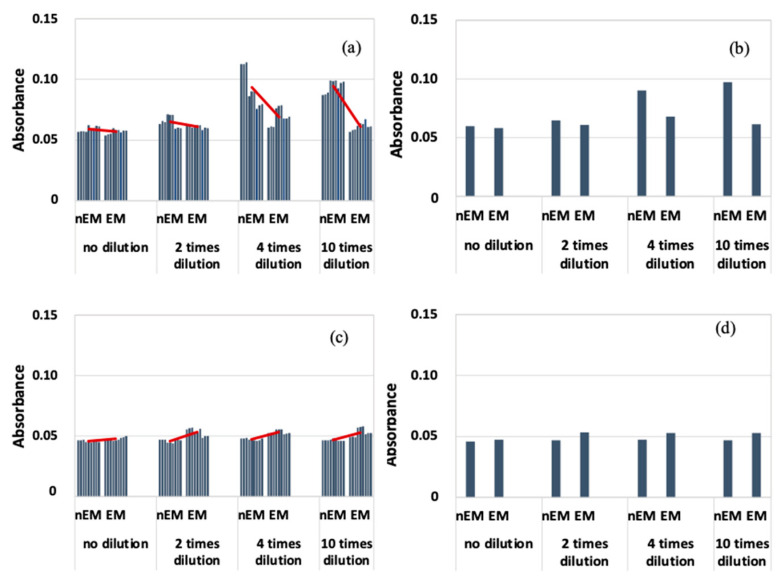
Absorbance measurement results during biofilm staining with *E. coli*; (**a**) All data when 20 kHz AC electromagnetic field was applied. (**b**) Median data when 20 kHz AC electromagnetic field was applied. (**c**) All data when 30 kHz AC electromagnetic field was applied. (**d**) Median data when 30 kHz AC electromagnetic field was applied.

The results of the absorbance measurement of the *E. coli* biofilm are shown in Figure 7, with absorbance on the vertical axis. 

Figure 7a shows that the absorbance of EM was lower than that of nEM under all dilution conditions at a frequency of 20 kHz. Because of the large variation in absorbance, the median absorbance values obtained under each dilution condition were calculated and compared with those of nEM and EM (Figure 7b). Again, the absorbance of EM decreased compared to that of nEM. On the other hand, at a frequency of 30 kHz (Figure 7c,d), the absorbance of EM showed a slight increase compared to nEM, and the same trend was confirmed when comparing median values. The obtained absorbance corresponds to the amount of biofilm formation. Therefore, *E. coli* biofilm was suppressed by the 20 kHz AC EM field but not by the 30 kHz field.

#### 3.2.2. Biofilm by *S. epidermidis*

**Figure 8:**
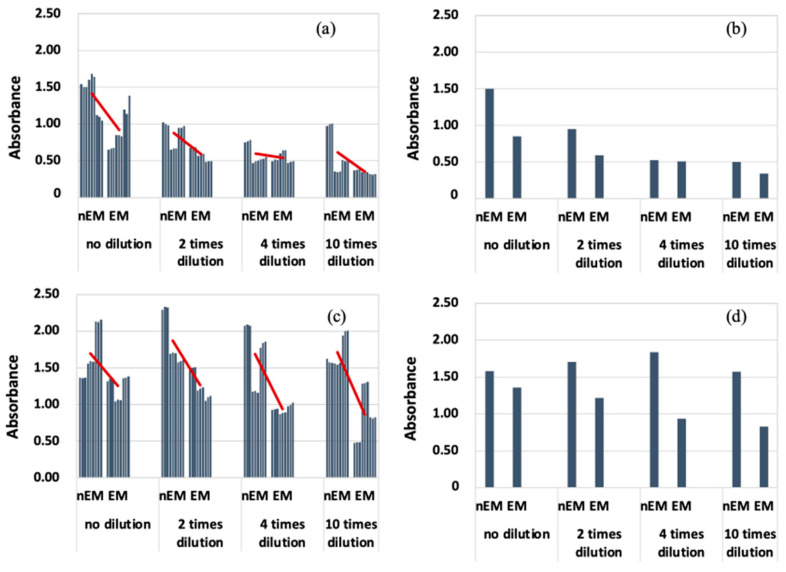
Absorbance measurements during biofilm staining by *Staphylococcus epidermidis*. (**a**) All data when 20 kHz AC electromagnetic field is applied. (**b**) Median data when 20 kHz AC electromagnetic field is applied. (**c**) All data when 30 kHz AC electromagnetic field is applied. (**d**) Median data when 30 kHz AC electromagnetic field is applied.

The results of absorbance measurement of the biofilm with *S. epidermidis* are shown in Figure 8. The median absorbance of EM was calculated for each dilution condition as in the case of *E. coli*, because the absorbance of *S. epidermidis* was highly variable. Therefore, the absorbance of EM was reduced compared to that of nEM under all dilution conditions. Therefore, the biofilm caused by *S. epidermidis* is suppressed by the AC electromagnetic field at both 20 and 30 kHz.

Comparing Figure 7 and Figure 8, the absorbance of *S. epidermidis* tended to be significantly higher than that of *E. coli*, i.e., the degree of staining was greater. There are two possible reasons for this difference. One is that the ability of *S. epidermidis* to form biofilms is higher than that of *E. coli*. The other is that the characteristics of Gram-negative and Gram-positive bacteria may be closely related. Gram-positive bacteria, i.e., epidermal staphylococci, have a thicker peptidoglycan layer, which allows them to retain crystal violet. This characteristic of the bacteria causes the absorbance of the samples after biofilm formation experiments (using *S. epidermidis*) to be higher. Therefore, it cannot be said that the biofilm-forming ability of *S. epidermidis* is higher than that of *E. coli*. It is difficult to measure the biofilm-forming ability among bacteria with this evaluation method. The reason why biofilm formation is suppressed when an AC electromagnetic field is applied is that some of the biofilm components absorb energy by absorbing the electromagnetic field. The energy that cannot be released at this time is converted into heat. Figure 7c,d shows that the biofilm suppression effect was not observed. The combination of bacteria and frequency means that the EPS components produced by each bacterium are different. Therefore, the EPS component of the biofilm produced by *E. coli* did not absorb the 30 kHz AC electromagnetic field, suggesting that the AC electromagnetic field had no biofilm suppression effect. Therefore, it was found that the EPS component of the biofilm and its electromagnetic field absorption band had a suppressive effect on biofilm formation. In the future, clarification of the EPS component of the BF for each bacterium will enable the selection of a frequency that has a biofilm suppression effect, which can be used to develop and apply the technology for practical use.

## 4. Discussion

The researchers have studied the effect of AC electromagnetic fields on biofilm suppression by injecting magnetic flux densities of up to 0.1 mT into material surfaces and varying the frequency around several kilohertz. Compared to conventional biofilm suppression methods, our series of studies on the application of alternating electromagnetic fields is expected to be safe due to the low magnetic flux density. There is the possibility of non-contact control, and it is also highly cost-effective. At first, the authors focused on more practical systems. The target was not a specific bacterium, but rather an environmental commensal bacterium in a laboratory atmosphere.

**Figure 9:**
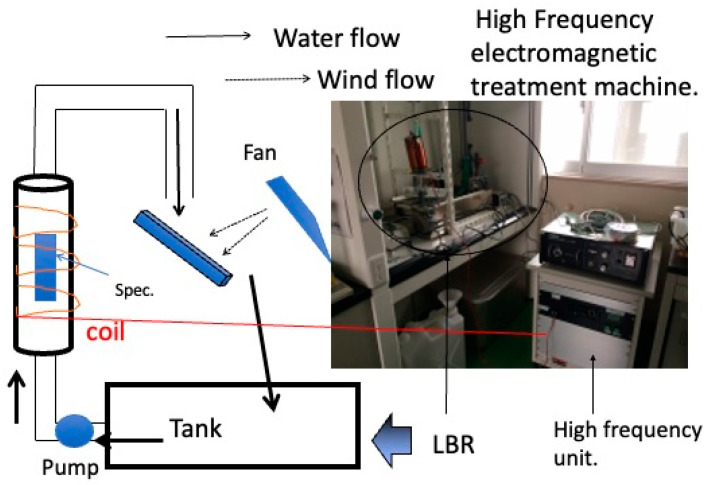
The laboratory biofilm reactor to investigate the effect of alternating electromagnetic fields on biofilm formation by environmental biota.

Initially, the target was not specific bacteria, but environmental commensal bacteria in a laboratory atmosphere. As shown in Figure 9, a biofilm-forming apparatus with a flow inlet was constructed, and purified water was circulated through the apparatus for a certain amount of time to form a biofilm on the surface of materials loaded in the apparatus. The specimens were loaded into a vertical column through which purified water was pumped. In the middle of the system, the purified water was released into the atmosphere and fell onto an intermediate plate, where it was mixed with bacteria from the atmosphere and fell into a tank. This process was repeated to continuously carry bacteria to the specimen surface, forming a biofilm. Biofilms produce various results depending on the combination of material and environment. The researchers conducted experiments on copper, aluminum, and carbon steel, which are practical metal materials, with the aim of developing countermeasures against biofilm scale formation in various types of piping. In all cases, they applied frequencies in the few kilohertz range they were aiming for. The 1 mT results showed that these results were effective at some frequencies and ineffective at others. In both cases, good results were obtained at 4 kHz or 8 kHz. In contrast to the flow system described above, experiments were conducted with *Escherichia coli* and *Staphylococcus epidermidis* in a static system with materials immersed in wells, as in the present experiment. In the case of conventional metallic materials, dissolution of metal ions from the surface of the material and the subsequent formation of corrosion products may occur. The magnetic flux density applied to the static system was 1 mT. In this case, no clear effect was obtained at 4 kHz, but the effect was obtained at 8 kHz for *Escherichia coli* and at 10 kHz for *Staphylococcus epidermidis*. These discrete effects are caused by the irreversible absorption of electromagnetic waves by the biofilm component polymers (the bacteria themselves or the EPS produced by the bacteria). The authors believe that these discrete effects are due to the irreversible absorption of electromagnetic waves by the biofilm component polymers, the bacteria themselves, or the EPS produced by the bacteria. The authors believe that the latter is the main factor, and that the energy of the biofilm inhibits biofilm formation in some form. Table 1 summarizes these results.

**Table 1:** The previous results for the application of alternating electromagnetic field to biofilms.

Bacteria	Materials	Flux Density	Effective Frequencies	References
Environmental biota	Pure copper	1 mT–5 mT	4 kHz, 8 kHz	[50,51]
Environmental biota	Pure aluminum	1 mT–5 mT	4 kHz, 8 kHz	[50,51]
Environmental biota	Carbon steel	1 mT–5 mT	4 kHz, 8 kHz	[50,51]
Environmental biota	Silane coated glass	1 mT	4 kHz	[52]
Environmental biota	Pure iron	1 mT	8 kHz	[38]
Environmental biota	Pure copper	1 mT	8 kHz	[38]
Environmental biota	Pure aluminum	1 mT	8 kHz	[38]
*E. coli*	Glass	1 mT	8 kHz, 16 kHz	[53]
*S. epidermidis*	Glass	1 mT	10 kHz	[54]

The effects of electromagnetic fields have recently been examined from a variety of perspectives. For example, Haagensen et al. [55] have obtained interesting results from experiments with smaller flux densities and lower frequency ranges. Cample et al. [56] performed experiments at the same magnetic flux density around 1 mT as the authors, but in a lower frequency range. They obtained some fruitful results. There are several points to consider regarding biofilm suppression by AC electromagnetic fields. Magnetic flux density is one of them, but the frequency range is also an important factor. Electromagnetic fields take various forms depending on the frequency. Ultraviolet rays are one of them, and X-rays and gamma rays are also electromagnetic waves, but their energies are very different. When electromagnetic waves of several to several tens of hertz are applied, the effects on bacteria and inorganic components in the environment are more likely to be in contrast. On the other hand, we focused on EPS, the extracellular polymerized substance produced by bacteria rather than on the bacteria themselves, because EPS forms the backbone of biofilms. We speculated that when electromagnetic waves are absorbed, the polymers that make up the EPS can be denatured, although the energy at this frequency is not sufficient to destroy them. Therefore, we considered the possibility that the biofilm may mechanically rupture or collapse without being able to maintain its skeleton. The evidence for this is the results of studies on the interaction between polymers and electromagnetic waves. These are summarized in Schnabel’s book [57]. One is that irreversible energy absorption occurs discretely (at some discontinuous and specific frequencies) by many highly polarized polymers, and indeed, results for many polymers indicate that highly polarized polymers can absorb energy in the frequency range from a few kilohertz to several tens of kilohertz. Another feature is that irreversible absorption occurs in the frequency range from a few kilohertz to several tens of kilohertz for highly polarizable polymers. Comparing these results with those of the present study, the similarity is high. We suspect that this is the main reason for the biofilm suppression in the experiments performed under the conditions of the present study.

In this experiment, the frequency of the alternative was increased to 20 kHz and 30 kHz, as in this experiment, the suppression effect of the AC electromagnetic field was observed for *S. epidermidis* at both 20 kHz and 30 kHz. However, for *E. coli*, as in previous experiments, it was either suppressed or not depending on the frequency. The inhibition of *E. coli* was observed at both 20 kHz and 30 kHz. Initially, we expected that the fluidization of the bacterial solution would impact the surface of the material to detach the biofilm, but this was not the case; the frequency that suppressed the biofilm was selective, as in the experiments using lower frequencies. In this respect, the results may not differ significantly from those obtained with the lower frequencies. However, we believe that our experiments and results in the higher frequency range are new findings in the following respects. One is the effect of the type of polymer on the absorption of electromagnetic waves. By increasing the frequency of the superimposed electromagnetic field from several kilohertz to several tens of kilohertz, it is thought that the number of polymers with the potential to absorb more electromagnetic waves has increased. The second is that, as in the case of the IH rice cooking described in the introduction, the water movement is more intense, and its physical impact effect on the biofilm is somewhat additional to the effect of the water movement.

Recently, an international standard for evaluating anti-biofilm materials has been adopted (ISO 4768:2023) [58] A measurement method for anti-biofilm activity on plastic and other non-porous surfaces has been established by the SIAA [59], composed of about 1200 and more antimicrobial-related companies that have been working with the authors. According to this standard, the evaluation system for biofilm evaluation is focused on the early logarithmic growth phase, around 10^3^ CFU/mL. This is because experiments conducted from the early logarithmic growth period more clearly show that polymers are produced by quorum sensing through the growth of bacteria on the material’s surface. The point of this new international standard is that, from a practical point of view, the quantification of biofilms can be undertaken by staining with crystal violet, rather than by instrumental analysis such as Raman spectroscopy or confocal laser microscopy, which gives more pre-bell quantitative results. For this reason, crystal violet was used for quantitative evaluation in this experiment. However, we started from a point where the concentration of bacteria in the experimental system was relatively high and just before the full growth state. In this regard, we believe that it will be important for future work to investigate the state when starting from a lower concentration to see the effect of the AC electromagnetic field. In addition, we believe that it is urgently necessary to investigate another angle to verify the effects of liquid flow and polymer absorption as described above. Applying AC electromagnetic fields at such bacterial concentrations will further examine this hypothesis and will be our next subjects for future study.

## 5. Conclusions

Glass specimens were immersed in a bacterial solution consisting of *Escherichia coli* and *Staphylococcus epidermidis*, and high-frequency AC electromagnetic fields of 20 kHz and 30 kHz were applied to the glass specimens to investigate the possibility of biofilm inhibition. The following results were obtained.

(1) In the case of *E. coli*, biofilm suppression was observed when 20 kHz was applied but not when 30 kHz was applied.
(2) In the case of *Staphylococcus epidermidis*, biofilm suppression was observed at 20 kHz and 30 kHz.
(3) These results were similar at the stationary phase of bacterial concentrations (about 10^9^ CFU/mL) and in the second half of the log growth phase (about 10^7^ CFU/mL).
(4) The partial or total disruption of the extracellular membrane by the AC electromagnetic field may have caused localized antimicrobial activity and suppressed bacterial growth, thereby inhibiting the presence of biofilms.

## Data Availability

Not applicable.

## References

[1] López D., Vlamakis H., Kolter R. (2010). Biofilms. Cold Spring Harb. Perspect. Biol..

[2] Branda S.S., Vik Å., Friedman L., Kolter R. (2005). Biofilms: The matrix revisited. Trends Microbiol..

[3] Otto M. (2008). Staphylococcal biofilms. Bact. Biofilms.

[4] Bridier A., Briandet R., Thomas V., Dubois-Brissonnet F. (2011). Resistance of bacterial biofilms to disinfectants: A review. Biofouling.

[5] Percival S.L., Malic S., Cruz H., Williams D.W., Percival S.L. (2011). Introduction to biofilms. Biofilms and Veterinary Medicine.

[6] Lappin-Scott H.M., Costerton J.W. (2003). Microbial Biofilms (Biotechnology Research).

[7] Costerton J.W., Lappin-Scott H.M., Lappin-Scott H.M., Costerton J.W. (1995). Introduction to Microbial Biofilms. Microbial Biofilms.

[8] Flemming H.-C., Szewzyk U., Griebe T. (2000). Biofilms—Investigative Methods & Applications.

[9] Rozman U., Filker S., Kalčíková G. (2023). Monitoring of biofilm development and physico-chemical changes of floating microplastics at the air-water interface. Environ. Pollut..

[10] Zhang Z., Christopher G. (2016). Effect of particulate contaminants on the development of biofilms at air/water interfaces. Langmuir ACS J. Surf. Colloids.

[11] Su Z., Zhang S., Zhang W., Guo Z., Li F., Li X. (2023). *Enterobacter* sp. biofilm at the air-water interface promotes carbonate precipitation. Int. Biodeterior. Biodegrad..

[12] Wang Z., Morales-Acosta M.D., Li S., Liu W., Kanai T., Liu Y., Chen Y.-N., Walker F.J., Ahn C.H., Leblanc R.M. (2016). A narrow amide I vibrational band observed by sum frequency generation spectroscopy reveals highly ordered structures of a biofilm protein at the air/water interface. Chem. Commun..

[13] Liu W., Li S., Wang Z., Yan E.C., Leblanc R.M. (2017). Characterization of surface-active biofilm protein BslA in self-assembling langmuir monolayer at the air–water interface. Langmuir ACS J. Surf. Colloids.

[14] Münster U., Heikkinen E., Knulst J. (1997). Nutrient composition, microbial biomass and activity at the air–water interface of small boreal forest lakes. Hydrobiologia.

[15] Kobayashi K., Iwano M. (2012). BslA (YuaB) forms a hydrophobic layer on the surface of Bacillus subtilis biofilms. Mol. Microbiol..

[16] Boomer S.M., Noll K.L., Geesey G.G., Dutton B.E. (2009). Formation of multilayered photosynthetic biofilms in an alkaline thermal spring in Yellowstone National Park, Wyoming. Appl. Environ. Microbiol..

[17] Hacke S., Möbius D. (2004). Influence of active sites distribution on CaCO_3_ formation under model biofilms at the air/water interface. Mesophases, Polymers, and Particles.

[18] Henk M.C. (2014). Capturing air–Water interface biofilms for microscopy and molecular analysis. Part of the Methods in Molecular Biology Book Series.

[19] Pace J.L., Rupp M.E., Finch R.G. (2005). Biofilms, Infection, and Antimicrobial Therapy.

[20] Roberts A.E., Kragh K.N., Bjarnsholt T., Diggle S.P. (2015). The limitations of in vitro experimentation in understanding biofilms and chronic infection. J. Mol. Biol..

[21] Costerton J.W. (2001). Cystic fibrosis pathogenesis and the role of biofilms in persistent infection. Trends Microbiol..

[22] Høiby N. (2017). A short history of microbial biofilms and biofilm infections. Apmis.

[23] Hall-Stoodley L., Stoodley P. (2009). Evolving concepts in biofilm infections. Cell. Microbiol..

[24] Bjarnsholt T. (2013). The role of bacterial biofilms in chronic infections. Apmis.

[25] Ch’ng J.-H., Chong K.K., Lam L.N., Wong J.J., Kline K.A. (2019). Biofilm-associated infection by enterococci. Nat. Rev. Microbiol..

[26] Donlan R.M. (2001). Biofilms and Device-Associated Infections. Emerg. Infect. Dis..

[27] Tenke P., Kovacs B., Jäckel M., Nagy E. (2006). The role of biofilm infection in urology. World J. Urol..

[28] Kirketerp-Møller K., Zulkowski K., James G. (2011). Chronic wound colonization, infection, and biofilms. Biofilm Infect..

[29] Wu M.Y., Sendamangalam V., Xue Z., Seo Y. (2012). The influence of biofilm structure and total interaction energy on Escherichia coli retention by Pseudomonas aeruginosa biofilm. Biofouling.

[30] Pons L., Delia M., Bergel A. (2011). Effect of surface roughness, biofilm coverage and biofilm structure on the electrochemical efficiency of microbial cathodes. J. Biotechnol..

[31] Neu T.R., Manz B., Volke F., Dynes J.J., Hitchcock A.P., Lawrence J.R. (2010). Advanced imaging techniques for assessment of structure, composition and function in biofilm systems. FEMS Microbiol. Ecol..

[32] de Carvalho C.C., da Fonseca M.M.R. (2007). Assessment of three-dimensional biofilm structure using an optical microscope. BioTechniques.

[33] Stoodley P., Bolyle J.D., Lappin-Scott H.M. Influece of flow on the structure of bacterial biofilms. Proceedings of the 8th International Symposium on Microbial Ecology.

[34] Block M.S., Rowan B.G. (2020). Hypochlorous acid: A review. J. Oral Maxillofac. Surg..

[35] Sharma D., Misba L., Khan A.U. (2019). Antibiotics versus biofilm: An emerging battleground in microbial communities. Antimicrob. Resist. Infect. Control.

[36] Abney S., Bright K., McKinney J., Ijaz M.K., Gerba C. (2021). Toilet hygiene—Review and research needs. J. Appl. Microbiol..

[37] Lin L., Jiang W., Xu X., Xu P. (2020). A critical review of the application of electromagnetic fields for scaling control in water systems: Mechanisms, characterization, and operation. NPJ Clean Water.

[38] Kanematsu H., Umeki S., Hirai N., Miura Y., Wada N., Kogo T., Tohji K., Otani H., Okita K., Ono T. (2016). Verification of Effect of Alternative Electromagnetic Treatment on Control of Biofilm and Scale Formation by a new Laboratory Biofilm Reactor. Ceram. Trans..

[39] Miyao M. (2021). Coevolution of a premium segment and product innovation: A case study of the Japanese rice cooker market. Asia Pac. J. Mark. Logist..

[40] Ivleva N.P., Wagner M., Horn H., Niessner R., Haisch C.J.A.C. (2008). In situ surface-enhanced Raman scattering analysis of biofilm. Anal. Chem..

[41] Ivleva N.P., Wagner M., Horn H., Niessner R., Haisch C.J.A. (2009). Towards a nondestructive chemical characterization of biofilm matrix by Raman microscopy. Anal. Bioanal. Chem..

[42] Beier B.D., Quivery R.G.J., Berger A. (2010). Identification of different bacterial species in biofilms using confocal Raman microscopy. J. Biomed. Opt..

[43] Samek O., Al-Marashi J., Telle H. (2010). The potential of Raman spectroscopy for the identification of biofilm formation by Staphylococcus epidermidis. Laser Phys. Lett..

[44] Beier B.D., Quivey R.G., Berger A.J. Confocal Raman microscopy for identification of bacterial species in biofilms. Proceedings of the Frontiers in Biological Detection: From Nanosensors to Systems III.

[45] Millo D., Harnisch F., Patil S.A., Ly H.K., Schröder U., Hildebrandt P. (2011). In situ spectroelectrochemical investigation of electrocatalytic microbial biofilms by surface-enhanced resonance Raman spectroscopy. Angew. Chem. Int. Ed..

[46] Beier B.D., Quivey R.G., Berger A.J. (2012). Raman microspectroscopy for species identification and mapping within bacterial biofilms. AMB Express.

[47] Czamara K., Majzner K., Pacia M.Z., Kochan K., Kaczor A., Baranska M. (2015). Raman Spectroscopy of lipids: A review. J. Raman Spectrosc..

[48] Chao Y., Zhang T. (2012). Surface-enhanced Raman scattering (SERS) revealing chemical variation during biofilm formation: From initial attachment to mature biofilm. Anal. Bioanal. Chem..

[49] Larkin P., Larkin P. (2011). General Outline and Strategies for IR and Raman Spectral Interpretation. Infrared and Raman Spectroscopy: Principles and Spectral Interpretation.

[50] Kanematsu H., Hirai N., Miura Y., Itoh H., Kuroda D., Umeki S. Biofilm leading to corrosion on materials surfaces and the moderation by alternative electro-magnetic field. Proceedings of the Materials Science and Technology Conference and Exhibition 2013, MS and T 2013.

[51] Kanematsu H., Umeki S., Ogawa A., Hirai N., Kogo T., Tohji K. The cleaning effect on metallic materials under a weak alternating electromagnetic field and biofilm. Proceedings of the Ninth Pacific Rim International Conference on Advanced Materials and Processing (PRICM9).

[52] Kanematsu H., Sasaki S., Miura Y., Kogo T., Sano K., Wada N., Yoshitake M., Tanaka T. (2015). Composite coating to control biofilm formation and effect of alternate electro-magnetic field. Mater. Technol..

[53] Kanematsu H., Katsuragawa T., Barry D.M., Yokoi K., Umeki S., Miura H., Zimmerman S. (2019). Biofilm formation behaviors formed by *E. coli* under weak alternating electromagnetic fields. Ceram. Trans. (Adv. Ceram. Environ. Funct. Struct. Energy Appl. II).

[54] Kanematsu H., Miura H., Barry D., Zimmermann S. (2019). Effect of alternating electromagnetic field on extracellular polymeric substances derived from biofilms and its mechanism. Contrib. Pap. Mater. Sci. Technol..

[55] Haagensen J.A., Bache M., Giuliani L., Blom N.S. (2021). Effects of resonant electromagnetic fields on biofilm formation in Pseudomonas aeruginosa. Appl. Sci..

[56] Di Campli E., Di Bartolomeo S., Grande R., Di Giulio M., Cellini L. (2010). Effects of extremely low-frequency electromagnetic fields on Helicobacter pylori biofilm. Curr. Microbiol..

[57] Schnabel W. (2014). Polymers and Electromagnetic Radiation Fundamentals and Practical Application.

[58] (2023). Measurement Method of Anti-Biofilm Activity on Plastic and Other Non-Porous Surfaces.

[59] Society of International Sustaining Growth for Antimicrobial Articles, SIAA Home Page. https://www.kohkin.net/en_index.html.

